# Neuromyelitis Optica Spectrum Disorder and Anti-MOG Syndromes

**DOI:** 10.3390/biomedicines7020042

**Published:** 2019-06-12

**Authors:** Marco A. Lana-Peixoto, Natália Talim

**Affiliations:** CIEM MS Research Center, Federal University of Minas Gerais Medical School, Belo Horizonte, MG 30130-090, Brazil; talim.fono@gmail.com

**Keywords:** neuromyelitis optica spectrum disorders, anti-MOG syndrome, aquaporin 4-IgG, myelin oligodendrocyte glycoprotein, multiple sclerosis

## Abstract

Neuromyelitis optica spectrum disorder (NMOSD) and anti-myelin oligodendrocyte glycoprotein (anti-MOG) syndromes are immune-mediated inflammatory conditions of the central nervous system that frequently involve the optic nerves and the spinal cord. Because of their similar clinical manifestations and habitual relapsing course they are frequently confounded with multiple sclerosis (MS). Early and accurate diagnosis of these distinct conditions is relevant as they have different treatments. Some agents used for MS treatment may be deleterious to NMOSD. NMOSD is frequently associated with antibodies which target aquaporin-4 (AQP4), the most abundant water channel in the CNS, located in the astrocytic processes at the blood-brain barrier (BBB). On the other hand, anti-MOG syndromes result from damage to myelin oligodendrocyte glycoprotein (MOG), expressed on surfaces of oligodendrocytes and myelin sheaths. Acute transverse myelitis with longitudinally extensive lesion on spinal MRI is the most frequent inaugural manifestation of NMOSD, usually followed by optic neuritis. Other core clinical characteristics include area postrema syndrome, brainstem, diencephalic and cerebral symptoms that may be associated with typical MRI abnormalities. Acute disseminated encephalomyelitis and bilateral or recurrent optic neuritis are the most frequent anti-MOG syndromes in children and adults, respectively. Attacks are usually treated with steroids, and relapses prevention with immunosuppressive drugs. Promising emerging therapies for NMOSD include monoclonal antibodies and tolerization.

## 1. Introduction

Neuromyelitis optica spectrum disorders (NMOSD) and anti-myelin oligodendrocyte glycoprotein (anti-MOG) syndromes are immune-mediated inflammatory conditions of the central nervous system (CNS) that frequently involve the optic nerves and the spinal cord. Because of their clinical manifestations and habitual relapsing course, they are frequently confounded with multiple sclerosis (MS). Early and accurate diagnosis of these distinct conditions is very relevant as they have different therapeutic approaches. Even a more important reason is the observation that some agents used in MS treatment may be deleterious to patients with NMOSD [1,2,3].

Although NMOSD and anti-MOG syndromes share a number of clinical manifestations, they are independent nosological entities with distinct pathophysiological mechanisms and histopathological features [4,5,6,7].

Whereas NMOSD is most frequently associated with antibodies which target aquaporin-4 (AQP4), the most abundant water channel in the CNS, particularly expressed in the astrocytic processes at the blood-brain barrier (BBB) [8], anti-MOG syndromes result from damage to myelin oligodendrocyte glycoprotein (MOG), a membrane protein expressed on oligodendrocyte cell surfaces and on the outermost surface of myelin sheaths. Because of this particular location, MOG is a good antigen candidate for autoimmune demyelination [6,7,9,10,11]. Moreover, three fourths of NMOSD patients test positive for AQP4 antibody, while serum MOG antibody is only detected in a minority of seronegative AQP4-IgG NMOSD patients [12,13,14].

The lack of coexistence of AQP4-IgG and MOG-IgG in the serum of a same patient further suggests that AQP4-IgG NMOSD and anti-MOG syndromes are distinct diseases [15]. On the other hand, failure to identify either AQP4-IgG or MOG-IgG in a proportion of patients with NMOSD phenotype supports the view that other autoantibodies or factors may also play a role in NMOSD pathogenesis. In line with that, N-methyl-D-aspartate receptor-IgG and CV2/CRMP5-IgG have been described in association with NMOSD phenotype [16,17,18,19].

## 2. Neuromyelitis Optica Spectrum Disorders

The term *neuromyelitis optica* was introduced by Eugène Devic and Fernand Gault in 1894, who first recognized the association of amaurosis and myelitis as a new clinical entity. Devic [20] reported the case of a 45-year-old French woman who was seen at the Hôtel-Dieu hospital of Lyon because of an intractable headache and depression in addition to general asthenia. One month later, she developed urinary retention, complete paraplegia and blindness, and died few weeks later. Autopsy disclosed severe demyelinating and necrotic lesions extending 4–5 cm length in the lower thoracic and lumbar spinal cord. There was demyelination of the optic nerves, but a gross examination of the brain was unrevealing. In this paper, Devic emphasized the similarity of the pathological process involving the spinal cord and the optic nerves, named the syndrome “neuro-myélite optique”, or “neuroptico-myélite”, and discussed its relationship with MS. Fernand Gault, a disciple of Devic’s, reviewed in detail 17 cases of this condition in his doctoral thesis named “De la neuromyélite optique aiguë” [21]. The eponym “Devic’s disease” was suggested by Acchiote [22]. However, the association of myelitis and blindness had already been reported by other authors in the early and mid 19th century. The *case of Marquis de Causan*—known as the first description of this association by the French anatomist and pathologist Antoine Portal in 1803–1804—was characterized by relapsing myelitis followed by amaurosis and signs of brainstem involvement [23]. Other previously reported cases included those by Giovanni Battista Pescetto in 1844 [24], Christopher Mercer Durrant in 1850 [25], Jacob Augustus Lockhart Clarke in 1862 [26], Thomas Clifford Albutt in 1870 [27], and Wilhelm Heinrich Erb in 1879–1880 [28]. Also, in the American continent, the association of optic neuritis and myelitis was identified by Seguin (1880) [29] prior to Devic and Gault’s pioneering publication. None of these previous authors however, had used the term “neuromyelitis optica”, or considered their cases as expression of a new nosological entity. It was only in 1943 that the disease was first identified in Latin America, when Aluizio Marques, in Rio de Janeiro, described two female patients who developed bilateral blindness and acute transverse myelitis [30].

### 2.1. Pathophysiology

The discovery of NMO-IgG and AQP4 as its targeted antigen unequivocally confirmed neuromyelitis optica as a disease distinct from MS and allowed its early laboratorial recognition [31,32]. The serum identification of AQP4-IgG expanded the clinical spectrum of the disease to include its limited forms (single or recurrent longitudinally extensive transverse myelitis [LETM], defined by MRI as a lesion extending for three or more vertebral segments, or recurrent isolated optic neuritis) [33], along with a wide variety of brainstem, diencephalic, and cerebral manifestations [34,35].

Aquaporin-4 monomers assemble to form tetramers which further aggregate in cell plasma membranes to form supramolecular arrays called orthogonal arrays of particles (OAP). There are two major forms of AQP-4: the full-length 323 amino acid M1 isoform, and the shorter 301 amino acid M23 isoform. Only the M23 isoform forms large OAPs [14]. It has been shown that M1 isoform does not form OAPs on its own, but can co-assemble with M23 in heterotetramers that limit OAP size [36,37].

Aquaporin-4 is widely expressed throughout the CNS. It is also highly expressed in the optic nerves and spinal cord, explaining their preferential involvement in the disease. Other CNS sites expressing AQP-4 include the supraoptic nucleus of the hypothalamus, the periventricular structures such as area postrema and the vascular organ of lamina terminalis, which lack BBB and contain osmo-sensitive neurons that regulate fluid homeostasis and release arginine-vasopressin, which facilitates this process. Aquaporin-4 is also expressed in non-neural tissues including skeletal muscle cells, lung airway cells, gastric parietal cells, renal collecting duct cells, inner ear, retinal Muller cells, lacrimal gland and salivary duct cells, and olfactory epithelial cells [38]. Human and experimental studies have shown that AQP4-IgG belongs mainly to IgG1 class, a potent activator of complement. The antibody enters the CNS, binds the antigen at astrocyte processes, induces complement-mediated inflammation, granulocyte infiltration, and astrocyte death [37,39]. Complement-mediated inflammation with secondary neutrophils and eosinophil infiltration plays a key role in the pathophysiology of NMOSD attacks [40].

Aquaporin-4 antibodies are more abundant in the peripheral blood than in the cerebrospinal fluid (CSF) [41]. In the periphery, they are produced by a number of B cell subpopulations which are vulnerable to interleukin-6 (IL-6), which enhances their survival and AQP4-IgG secretion [42]. Although AQP4-IgG can gain direct access to AQP4 on astrocytes located at circumventricular organs where the endothelia lack tight junction, the mechanisms of its penetration into other CNS sites that are protected byBBB are still unclear. Recent findings have shown that AQP4-IgG is not sufficient or even necessary to cause BBB disruption [43]. Sera from NMO patients contain non-reactive AQP4 antibodies, identified as recombinant antibodies (rAb) ON-12-2-46 and ON-07-5-31 which target glucose-regulated protein 78 (GRP78) on the cell surface of brain microvascular endothelial cells (BMEC). GRP78 is a stress protein of the heat shock 70 family expressed in all CNS cells [44]. However, only rAb ON 12-2-46 induces nuclear translocation of nuclear factor kB (NF-kB) p65, which is a marker of cell activation [43]. BMECs activation causes increased secretion of vascular endothelium growth factor (VEGF) and metaloproteinases (MMP)-2/9 which result in a down regulation of claudin 5 and disruption of the BBB. Leakage of the BBB allows entrance of AQP4-IgG to the CNS and its binding to AQP4 in the astrocytic endfeet. Evidences showing the causal role of AQP4-IgG in NMOSD include its nearly absolute specificity for the disease; its correlation with disease activity, higher number of relapses and more severe course as compared with seronegative patients; some distinct demographic and clinical features; increased concentration of AQP4-positive plasmablasts in NMOSD patients, mainly during disease relapses; and decreased serum AQP4-IgG concentration following successful treatments and during disease remission [45,46,47]. Histopathological features such as a marked loss of astrocytes and accumulation of IgG and IgM around blood vessels, the site of AQP4 expression; spare of myelin and axons in some lesions suggesting that astrocytes (which have a higher expression of AQP4) are the initial cell target in the disease, whereas in more recent lesions there may be preservation of glial fibrillary acidic protein (GFAP), a marker of astrocyte damage, suggesting that AQP4 is the primary target of the immune attack. The initial loss of AQP4 with astrocyte preservation might reflect the internalization of AQP4 of either M1-AQP4 or both isoforms before a complement becomes locally available to mediate a lytic inflammatory process. This would account for the rapid reversal of some MRI abnormalities in the area postrema and some parts of the cerebrum. The disease individual clinical phenotype and severity may be related to the ratio of AQP4-M1 to M23 in the optic nerves, brain, and spinal cord. While CNS regions with a higher proportion of M1 would rapidly internalize, avoiding a cascade of tissue damage, areas richer in AQP4-M23 isoform would be more liable to necrotic lesions and cavitation [44,45,46].

Additionally, following a decreased serum concentration of AQP4-IgG, the AQP4-M1 isoform is rapidly replaced in the astrocytic membrane. Experimental studies have showed that a passive transfer of AQP4-IgG from NMOSD patients to animals with disrupted BBB by previous experimental autoimmune encephalomyelitis (EAE), or through pretreatment with Freund’s adjunct, develop CNS typical NMO histopathological lesions [37,48,49,50,51].

### 2.2. Epidemiology

Some caveats are needed before looking at published data on the epidemiology of NMOSD. First, studies on the frequency and distribution of the disease across the world are still scanty and most of them are based on small cohorts. Additionally, they employed non-standardized methodology and inconsistent inclusion of seronegative patients, and differ regarding used diagnostic criteria and assays for AQP4 antibodies detection. In spite of these limitations, a number of issues related to disease prevalence, ethnicity and geography have been clarified. Studies in different populations and geographic regions show that NMOSD is a rare disorder with worldwide distribution [52]. However, an exception to these studies is the seroprevalence study from Olmsted County, in the United States, and Martinique [53] which showed a 2.5-fold higher prevalence rate of the disease in Martinique (10 per 100,000) than in Olmsted County (3.9 per 100,000), all other studies [52] indicated a fairly uniform prevalence rate below 5 per 100,000 people in different regions and populations (Table 1). Likewise, incidence rates were also homogeneous in different countries, ranging from 0.2 per 1 million per year, in Mexico [54] to 4.0 in Denmark [55]. Although most studies point to incidence rate below 1 per 1 million, a peak incidence rate of 7.3 per 1 million was again found in Martinique [53].

Although NMOSD has been regarded to have a predilection for non-Caucasians, the similarity of prevalence and incidence rates in different geographic regions and distinct ethnicities may suggest that, as in opposition to MS, latitude and genetic factors may not play a key role in NMOSD pathogenesis [70]. This contradicts the ethnicity-specific higher prevalence for blacks in Olmsted County, which was similar to that found in the black population of Martinique [53]. The Australian-New Zealand study also observed higher prevalence rate in people with Asian ancestry than in Caucasians [67].

Ethnicity, however, influences age at onset and phenotype of AQP4 seropositive NMOSD. A recent study comparing the clinical manifestations and outcome of 603 NMOSD patients of three different races (Asians, Caucasians and Afro-Americans/Afro-Europeans) showed that non-white patients are younger at disease onset, and more frequently have brain attacks at onset or during the disease course, as well as more frequent abnormalities on brain MRI. Afro-American and Afro-European patients have more severe attacks at onset than Asians and Caucasians, but the outcome at the last follow-up was similar in the different racial groups [71]. This observed that the similarity of the outcome at the last follow-up for all racial groups is in opposition to previous reports of a more severe outcome of NMOSD in Afro-Caribbean than in Caucasian patients [56,72,73].

Usually, the initial clinical manifestations of NMOSD occur at an age of around 35-45 years (median age at onset is 39), but children and the elderly account for 18% of cases. Women comprise 70% to 90% of all cases, but there is no gender predilection in children [74]. The estimated proportion of familial cases (3%) is greater than expected based on the disease prevalence [75]. In some populations, human leukocyte antigen (HLA) has been reported to be associated with susceptibility to NMOSD, such as HLA-DPB1*0501 allele in Japanese and Chinese populations [76,77] and HLA-DPB1*03 in Caucasian, Afro-Caribbean, and Indian patients [78,79,80,81]. These genetic factors may account for the phenotypic variability among racial groups.

### 2.3. Clinical Manifestations

For a long time, the hallmark of NMOSD had been considered as the preferential involvement of the optic nerves and the spinal cord in absence of brain symptoms. However, following the discovery of NMO-IgG in 2004, a wide variety of brainstem, diencephalic and cerebral signs were described in seropositive patients [34]. In 2015 the International Panel for NMO Diagnosis (IPND) added area postrema, brainstem, diencephalic and cerebral manifestations to optic neuritis and LETM to the revised diagnostic criteria for NMOSD [35]. Table 2 shows the clinical manifestations of NMOSD. Clinical analysis of the largest international cohort of AQP4-seropositive NMOSD so far published [71] shows that the disease was relapsing in 85% of the cases. Myelitis was the initial manifestation in 48%, optic neuritis in 42%, area postrema syndrome in 10%, brainstem/diencephalic/cerebral symptoms in 14%, and simultaneous optic neuritis and myelitis in 4%. During the disease course, 84% of the patients presented myelitis, 63% optic neuritis, 15% APS, 17% brainstem syndrome, 3% diencephalic syndrome and 14% cerebral syndrome. In almost one half of the patients (45%) the inaugural attack was severe (defined as an Expanded Disability Status Scale (EDSS) score at ≥6.0 or visual acuity ≤0.1 in at least one eye at nadir).

Severe transverse myelitis in NMOSD most commonly causes symmetrical motor and sensory loss, mainly of the lower limbs, associated with sphincter disturbances. Hiccups and respiratory failure may result from an extension of cervical lesions to the medulla oblongata. Intractable nausea, vomiting and hiccups indicate involvement of the area postrema. The area postrema is the chemosensitive vomiting center located in the dorsal part of the medulla oblongata. It is highly vascularized, lacks blood brain barrier and has a high AQP-4 expression. Its fenestrated capillaries and loosely apposed astrocytic processes likely facilitate IgG access to the CNS [82]. This increased exposure to AQP4-IgG may explain the frequent occurrence of incoercible nausea/vomiting/hiccups in AQP4-IgG seropositive patients. Cervical lesions with rostral extension to the area postrema as seen on MRI, have been observed in other conditions, such as sarcoidosis, lymphoma, paraneoplastic myelitis, spondylosis and dural arteriovenous fistula. However, area postrema lesions on MRI occurring in association with incoercible nausea, vomiting or hiccups are specific for NMOSD [83].

Optic neuritis in NMOSD may differ from isolated idiopathic optic neuritis, and from optic neuritis occurring in MS. In NMOSD, optic neuritis is characterized by more severe visual loss at onset, bilateral involvement of the optic nerves or optic chiasm, relapsing course, poor response to IV corticosteroid pulses, poor recovery with permanent visual deficits, and association with normal brain MRI, or with unspecific lesions on brain MRI. Bitemporal hemianopsia points to the presence of chiasmal involvement, which is more common in AQP4-IgG NMOSD than in MS or anti-MOG syndromes.

Brainstem symptoms occur in about one third of the NMOSD patients and are the inaugural manifestation of the disease in about one half of these cases. The most commonly observed brainstem symptoms are vomiting (33%), hiccups (22%), oculomotor dysfunction (20%), and pruritus (12%), followed by hearing loss, facial palsy, vertigo, and trigeminal neuralgia (about 2% each) [84].

In a study of a multi-racial cohort, associated systemic autoimmune diseases were observed in 30% of the Caucasian, 9% of Asian, and 19% of the Afro-American/Afro-European AQP4-seropositive patients [71]. Serum autoantibodies associated with autoimmune conditions are frequently found in NMOSD patients, even in the absence of clinical manifestations. The most common autoimmune abnormalities associated with NMOSD include those related to thyroid disease, systemic lupus erythematosus, and Sjögren syndrome [46].

### 2.4. Laboratorial Characteristics

Patients with suspected NMOSD need a careful history and physical examination followed by a comprehensive laboratory work-up to rule out mimickers. Laboratory evaluation should include tests for infectious diseases, sarcoidosis, lymphomas and other tumors, paraneoplastic disorders, metabolic and nutritional disorders as well as a number of other autoimmune conditions. Testing for serum AQP4-IgG and MOG-IgG has diagnostic relevance for all patients with suspected NMOSD. Previously employed techniques for serum detection of AQP4-IgG such as indirect immunofluorescence and enzyme-linked immunosorbent assay (ELISA) proved to have lower sensitivity and specificity than cell-based assays (CBA) (mean sensitivity of indirect immunofluorescence and ELISA were 63% and 64%, respectively). Moreover, ELISA may yield 0.5%–1.2% of false-positive results [85,86,87]). There is a marked variation in assay sensitivity, which ranges from 48.7% to 76.7%, with the highest sensitivity obtained with CBA [88]. The specificity of the different assays ranged from 86.9% to 100% with the commercial fixed CBA having higher specificity than the live CBA. False negative results are higher during remissions, after plasma exchange, or in use of immunosuppressive drugs [88].

Recommendations for testing for serum AQP4-IgG include: (1) patients with acute transverse myelitis associated with a LETM lesion on spinal MRI, or with myelitis associated with normal brain MRI or without evidences of MS or other causes; (2) patients with optic neuritis with atypical features, such as the occurrence of relapses, bilateral simultaneous involvement of the optic nerves or chiasmal involvement, poor recovery, or optic neuritis associated with a long lesion of the optic nerve; (3) patients with area postrema syndrome; (4) patients with diencephalic symptoms and MRI abnormalities of unknown etiology; and (5) patients with encephalopathy of unknown nature. Testing for AQP4-IgG is not recommended in patients with typical clinical and imaging evidences of MS [14,88].

Cerebrospinal fluid (CSF) analysis usually discloses distinct features from those found in MS. While oligoclonal bands (OCB) restricted to the CSF occur in more than 90% of the MS population [89], they were found in only 18% of a large NMOSD cohort [90]. Interestingly, OCB restricted to the CSF are less frequently observed in Asian than in Caucasian or African-American/African European NMOSD patients [71]. During acute relapses a variable pleocytosis with presence of neutrophils and eosinophils may be observed [46].

### 2.5. Diagnostic Criteria

Current diagnostic criteria for NMOSD were developed by the IPND in 2015 [35]. The panel took the following decisions: (1) unify NMO and NMOSD under the single term “NMOSD”; (2) define (i) optic neuritis; (ii) acute myelitis; (iii) area postrema syndrome or episode of otherwise unexplained hiccups or nausea and vomiting; (iv) acute brainstem syndrome; (v) symptomatic narcolepsy or acute diencephalic clinical syndrome with NMOSD-typical diencephalic MRI lesions; and (vi) symptomatic cerebral syndrome with NMOSD-typical brain lesions as the six “core clinical characteristics” of NMOSD, according to involvement of anatomic sites; (3) establish diagnostic criteria for both NMOSD with AQP4-IgG and NMOSD without AQP4-IgG (negative serology or not performed test) (Table 3); (4) require additional supportive MRI characteristics to diagnostic criteria for NMOSD without AQP4-IgG or with unknown AQP4-IgG in order to enhancing specificity; (5) recommend the use of CBA for AQP4-IgG detection due to their higher sensitivity and specificity; (6) list some clinical features as well as laboratory and imaging findings that may point to alternative diagnoses, and therefore must be seen as “red flags”.

Table 3 shows the international consensus diagnostic criteria for NMOSD with AQP4-IgG, and the diagnostic criteria for NMOSD without AQP4-IgG or with AQP4-IgG unknown status. For AQP4-IgG seropositive individuals, at least one of six “core clinical characteristics” must be present. For individuals without AQP4-IgG or with unknown AQP4-IgG status, diagnosis of NMOSD requires at least two of the six core clinical characteristics. One of the six core clinical characteristics must be optic neuritis, transverse myelitis or area postrema syndrome, and all of them need additional supportive MRI characteristics.

Clinical signs, CSF, MRI and optic coherence tomography (OCT) findings usually distinguish NMOSD from MS. Most frequently, atypical features for NMOSD (“red flags”) point to the diagnosis of MS (Table 4). However, a number of other conditions may mimic NMOSD by involvement of the optic nerves and/or the spinal cord (Table 5) [91].

### 2.6. Magnetic Resonance Imaging

Magnetic resonance imaging of the brain and spinal cord is an essential tool for the diagnosis and management of demyelinating diseases of the CNS. The correct differentiation of NMOSD and anti-MOG syndromes from MS is important to provide patients with the most appropriate treatment.

**Figure 1:**
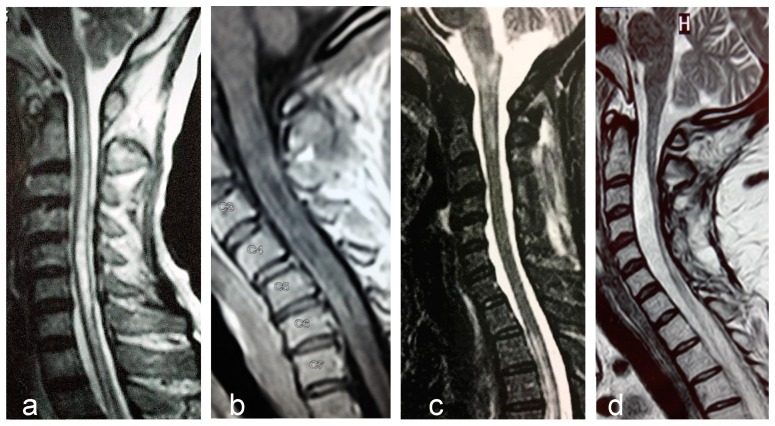
Examples of longitudinally extensive spinal cord lesions detected by MRI in AQP4- seropositive NMOSD patients. (**a**). T2-weighted central longitudinally extensive cervical lesion. (**b**). T1-weighted lesion with gadolinium showing multiple hypointensities (cavitations) throughout the cervical cord. (**c**). T2-weighted cervical lesion extending to brainstem. Another lesion is seen in the upper thoracic levels. (**d**). Longitudinally extensive spinal cord atrophy of the cervical cord.

Longitudinally extensive transverse myelitis, is the most specific imaging feature of NMOSD (Figure 1a). The length of the lesion has been considered the most distinguishing feature from MS, although long lesions may occur in MS and short lesions in NMOSD. Frequently, LETM lesions exhibit non-homogeneous contrast-enhancing that may persists for months following acute attacks. An extensive centrally-located hypointense signal in T1-sequence denotes cavitation secondary to tissue necrosis (Figure 1b). Cervical lesions may extend rostrally to the medulla oblongata (Figure 1c). Longitudinally extensive cord atrophy results from severe or recurring myelitis (Figure 1d). Short lesions, characterized by extension < three vertebral segments have been reported, predominantly at disease onset in 14% of the patients [92].

**Figure 2:**
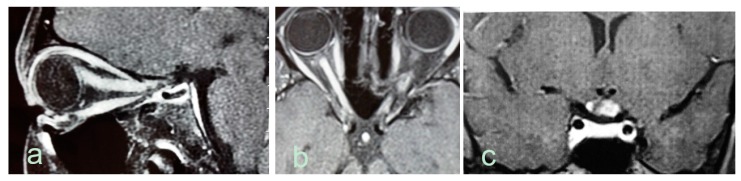
Optic nerve abnormalities on MRI in AQP4-seropositive NMOSD patients. (**a**). Sagittal T1-weighted MRI shows edematous gadolinium-enhancing optic nerve lesion extending from the eye to the intracranial segment. (**b**). Axial T1-weighted extensive gadolinium-enhancing lesion in both optic nerves. (**c**). Coronal T1-weighted MRI shows edematous gadolinium enhancing lesion in the optic chiasm.

Optic nerve abnormalities differ between, NMOSD and MS. Thickened, contrast-enhancing and long (≥ one-half the length of the optic nerve) lesions, as well as preference for involvement of the posterior segment of the nerve or chiasm are all in favor of NMOSD (Figure 2).

Most NMOSD patients have abnormalities on brain MRI [93]. More commonly, brain MRI lesions are unspecific, but they fulfill Barkoff’s criteria for MS in up to 42% of patients [93,94]. In a minority of cases, NMOSD typical brain lesions can be identified mainly in AQP4 enriched regions, such as around the lateral, third and fourth ventricles [93]. Brain lesions that favor NMOSD more than MS include peri-ependymal lesions surrounding the ventricles and aqueduct, hemispheric tumefactive lesions, extensive lesions involving corticospinal tracts, and “cloud-like” enhancing lesions [95]. One recent study [96] showed that criteria comprising (1) at least one lesion adjacent to the body of the lateral ventricle and in the inferior temporal lobe; or (2) the presence of an S-shaped U-fiber lesion; or (3) a Dawson’s finger type lesion were fulfilled by 90.9% RRMS, 12.9% AQP4-IgG NMOSD, and 4.8% MOG-IgG NMOSD patients.

Adults and children with MOG antibody disease frequently had fluffy brainstem lesions, often located in pons and/or adjacent to fourth ventricle. Children across all conditions showed more frequent bilateral, large, brainstem and deep grey matter lesions. MOG antibody disease spontaneously separated from multiple sclerosis, but overlapped with AQP4 antibody disease. Multiple sclerosis was discriminated from MOG antibody disease and from AQP4 antibody disease with high predictive values, while MOG antibody disease could not be accurately discriminated from AQP4 antibody disease. The best classifiers between MOG antibody disease and multiple sclerosis were similar in adults and children, and included ovoid lesions adjacent to the body of lateral ventricles, Dawson’s fingers T1 hypointense lesions (multiple sclerosis), fluffy lesions and three lesions or less (MOG antibody). In the validation cohort patients with antibody-mediated conditions were differentiated from multiple sclerosis with high accuracy [96].

### 2.7. Treatment

In spite of their clinical similarities, NMOSD and MS have different treatment. It has been shown that most MS disease modifying drugs, including beta-interferons, glatiramer acetate, natalizumab, alemtuzumab, fingolimod and dimethyl-fumarate are not only inefficacious in NMOSD, but may cause disease exacerbation [97].

In NMOSD the outcome of attacks is usually poor. Recent analysis of 871 attacks revealed that complete remission occurred in only 21% of them and 6% of them had no improvement [98]. The sequence of treatments is of fundamental importance to improve the outcome. Medical therapy, therefore, aims both to enfeeble an ongoing inflammatory attack, and avoid future relapses.

#### 2.7.1. Therapy of Acute Relapses

Relapses are usually treated with intravenous pulses of methylprednisolone (one gram/day for five days). In severe NMOSD attacks, or when corticosteroids fail to stabilize progression of symptoms plasma exchange (PLEX) must be added [99]. Apheresis eliminates the pathogenic antibodies, from circulation and has higher therapeutic efficacy than IV corticosteroids. Its early use as first-line therapy following attack is a predictor of better remission.

Post-infusion oral prednisone is usually recommended, mainly when an immunosuppressive agent with delayed onset of action is prescribed as prophylaxis of new events [100]. Azathioprine, mycophenolate mofetil, and rituximab are the most commonly used immunosuppressive treatments for prevention of new attacks of the disease.

#### 2.7.2. Therapy for Relapses Prevention

Table 6 shows the various drugs used for prevention of relapses in NMOSD. Prednisone, azathioprine, mycophenolate mofetil and rituximab are the first-line drugs. The choice of the initial treatment depends on availability, costs, co-morbidities, and disease course. Prednisone is inexpensive and has a rapid-onset therapeutic action, but adverse effects frequently restrain its continuation for a long time.

Azathioprine is probably the most commonly used drug in the preventive treatment of attacks in NMOSD. Initially, it should be combined with prednisone for three to six months until its maximal therapeutic effect can be reached. The lymphocyte count should decrease to 600–1000/cubic millimeter and the mean erythrocyte volume should increase five points from baseline. Thiopurine methyltransferase enzyme activity testing, when available is recommended before the administration of the drug to avoid higher risk of adverse effects. Monitoring of blood cell count and liver function tests on a regular basis is mandatory.

Mycophenolate mofetil is recommended as an alternative treatment in patients who develop intolerance or poor response to azathioprine.

Rituximab is a chimeric monoclonal anti-CD20 antibody that produces rapid depletion of circulating CD20 B cells. A number of studies have showed its efficacy and tolerance in the treatment of NMOSD, but some aspects of treatment strategy and long-term safety still remain to be clarified [101].

Monoclonal antibodies will probably play a most important role in treatment of NMOSD in the coming years. Eculizumab and tocilizumab have already shown their efficacy in small groups of patients [102].

Eculizumab is a humanized monoclonal antibody that inhibits the complement protein C5 and blocks terminal complement activation [103]. The complement cascade is a fundamental part in the inflammation process in NMOSD lesions. In spite of eculizumab efficacy in preventing relapses the increased risk of patients developing meningococcal meningitis raises important safety concerns [104].

Tocilizumab is a monoclonal antibody that targets Interleukin-6 (IL-6) receptor and decrease survival of the antibody-producing plasmablasts. Inebelizumab is a humanized anti-CD19 monoclonal antibody that targets B cell lineage. Although there is still no open-label study supporting its use, it is probably more efficacious than rituximab, which targets the more mature CD20. Inebelizumab removes plasmablasts that express CD19, decreasing the production of AQP4-IgG [102].

Satralizumab is an anti-IL-6 receptor monoclonal antibody. A recent communication on results of a phase III study showed that Satralizumab is a promising therapeutic agent by reducing the risk of relapses by 62% in NMOSD patients [105].

Tolerization is a recent therapeutic approach that uses innovative techniques to restore immune tolerance to host antigens and suppress autoimmune diseases [106]. Tolerization techniques include inverse DNA vaccination, T-cell vaccination, peptide-coupling strategies, tolerogenic dendritic cell vaccination, as well as T-cell receptor engineering-, and chimeric antigen receptor-based therapeutics. As AQP-4 is a specific target to NMO-IgG, there is reason for optimism that this new approach might offer marked beneficial to NMOSD patients, avoiding the wide variety of adverse effects of chronic immunosuppressive agents.

## 3. Anti-Myelin Oligodendrocyte Glycoprotein Syndromes

Myelin oligodendrocyte glycoprotein is a component of myelin expressed exclusively in myelin produced by oligodendrocytes in the CNS, making up less than 0.05% of total myelin proteins. It presents a length of 245 amino acids with a molecular weight of approximately 26–28 kDa [107,108,109].

The introduction of experimental autoimmune encephalomyelitis (EAE) as an animal model of demyelination raised the interest in the search of anti-MOG antibodies in MS patients. Some investigators reported a prevalence as high as 41% of anti-MOG antibodies serum positivity in MS patients [110]. Others, however, found similar rates of positive MOG-IgG serostatus inpatients with MS, other neurological disorders and healthy controls [111,112,113,114,115,116,117]. Recently, the introduction of CBA in substitution to enzyme-linked immunosorbent assays and immunoprecipitation techniques for the detection of MOG-IgG, methods which were not reliable, led to a major change in the understanding of the relationship between MOG-IgG and CNS disorders in humans. Using CBA, a technique that preserve the conformational structure of full-length human MOG, antibodies targeting MOG have been identified in both children and adults with a variety of phenotypes such as ADEM, optic neuritis, transverse myelitis, NMOSD, and brainstem encephalitis [118,119,120,121,122]. Conversely, MOG-IgG has rarely been found in patients with MS phenotype [123,124].

### 3.1. Pathophysiology

While the role of AQP4-IgG in the pathophysiology of NMOSD has been established by a large number of clinical and experimental evidences the innermost mechanisms underlying the variety of human demyelinating phenotypes in association with anti-MOG antibodies remain to be better clarified.

Anti-MOG antibodies are produced peripherally and usually reach the CNS following a breakdown of the BBB secondary to infections. A history of preceding infectious prodrome is reported in almost 50% of the patients [124]. The absence of restrict oligoclonal bands in the CSF of patients with anti-MOG syndromes supports the notion of its peripheral origin. Circulating lymphocytes may also migrate to CNS with subsequent clonal expansion [125].

Both in vivo and in vitro studies have suggested the presence of complement in mediating demyelination [126,127]. The observation of complement-mediated cytotoxicity from in vitro studies, and the development of a NMOSD-like disorder in animal models are strong evidences in favor of MOG-IgG pathogenicity [18,128,129].

However, in some instances there are reversible alterations to myelin without complement activation or inflammatory cell infiltration [130]. This is in consonance with the better recovery of some patients with anti-MOG syndromes as compared with NMOSD [119,131].

There are few pathological studies on anti-MOG syndromes [7,10]. A brain biopsy from a patient with MOG-antibody-associated encephalomyelitis revealed typical MS-type II histopathological features characterized by deposition of IgG and activated complement at sites of ongoing demyelination. There were well demarcated areas of loss of myelin with relative preservation of axons and astrocytes, numerous lipid-laden macrophages containing myelin debris, and inflammatory infiltrates with predominately perivascular T cells and some perivascular B-cells [7]. However, search for MOG-IgG and a number of other autoantibodies in a series of patients with Type-II MS failed to show any direct relation between type II-MS and MOG-IgG [6]. In contrast with seropositive AQP4-IgG NMOSD, co-existing serum autoantibodies are rare in anti-MOG syndromes. Associated autoimmune disorders are found in over one third of patients with AQP4-IgG seropositive NMOSD, but in only 9% of the anti-MOG syndromes.

### 3.2. Epidemiology

Major published series show that anti-MOG syndromes have an earlier age at onset, a lower female to male ratio, and a different racial predisposition as compared with seropositive AQP4-IgG NMOSD [96,119,124,132]. In a recent analysis of 50 cases [6], the age at onset ranged from 6 to 70 years (median 31 years) and 64% were females. Caucasians comprised 73% of the 59 patients in Australia/New Zealand series [124].

### 3.3. Clinical Manifestations

Almost all patients with anti-MOG syndromes present a relapsing course. The proportion of patients with a monophasic disease declines with extension of the follow-up. Relapses occurred in 93% of patients with disease duration ≥8 years [6]. In a study of 276 relapses in 50 patients, optic neuritis occurred in 88%, acute myelitis in 56%, brainstem attacks in 24%, supratentorial encephalitis in 14%, and cerebellitis in 4% of the patients. Bilateral simultaneous optic neuritis occurred in 51% and simultaneous optic neuritis and myelitis in 18 % of the patients [6].

Anti-MOG syndromes have distinct clinical features in children and adults. In children MOG-IgG most frequently expresses clinically as ADEM phenotype, whereas optic neuritis, usually with bilateral involvement, predominates in adults. In a study of 59 patients with relapsing anti-MOG syndromes (33 children and 26 adults) [124] the inaugural symptoms in the pediatric group were ADEM (36%), bilateral optic neuritis (24%), unilateral optic neuritis (15%). In adults, optic neuritis was the presenting symptom in 73% (bilateral optic neuritis 42%; unilateral optic neuritis 31%). Simultaneous involvement of the optic nerves and spinal cord (NMOSD phenotype was the presenting symptom in two children (6%) and five adults (19%). ADEM did not occur in the adult group. Transverse myelitis was less common. Conversely, myelitis occurred at disease presentation in 34% of the patients in another series [6], whereas optic neuritis in 74%, brainstem encephalitis in 8%, cerebral symptoms in 6% and cerebellar symptoms in 2%. At presentation, most patients exhibit either isolated optic neuritis (64%), isolated myelitis (18%), or combined optic neuritis and myelitis (10%). [6]. Optic neuritis is usually severe. Visual acuity ≥20/200 is observed in almost 70% of patients and optic nerve head swelling in the vast majority of the cases [124,132].

Interestingly, 25% to 32% of the patients in both series fulfilled the 2015 International consensus criteria for NMOSD, whereas 15% to 33% of them fulfilled revised McDonald criteria for MS [124,132].

### 3.4. Anti-MOG Testing

The recently introduced CBA techniques to detect specific autoantibodies that recognize conformational epitopes of membrane proteins, are the currently recommended method for the detection of AQP4-IgG and MOG-IgG. Indications for testing are based on the presence of specific clinical and paraclinical abnormalities that are considered typical for these disorders and atypical for MS. As some patients with MOG-related disorders may test negative for MOG-IgG during disease remission and treatment with immunosuppressive agents, it is recommended that the search for the antibody should be performed during acute relapses.

Many factors influence the sensitivity and specificity for anti-MOG antibody detection and the discrepancies found in early studies are now considered as a result of the use of inappropriate methodology for antibody detection, such as ELISA and immunoblot techniques. Using CBA antibodies targeting MOG have been recently identified in both children and adults with demyelination disorders including acute disseminated encephalomyelitis (ADEM), optic neuritis (ON), transverse myelitis (TM), and AQP4-seronegative NMOSD [124].

### 3.5. Cerebrospinal Fluid Analysis

Pleocytosis is found in over one half of patients with anti-MOG syndromes. White cell counts ≥100 cell/μL have been reported in 28% of cases [132]. Neutrophils may be present in variable proportion. Intrathecal IgG synthesis as measured by the presence of restricted oligoclonal bands in CSF was found in 11% to 13% of patients [124,132].

### 3.6. MRI Features

**Figure 3:**
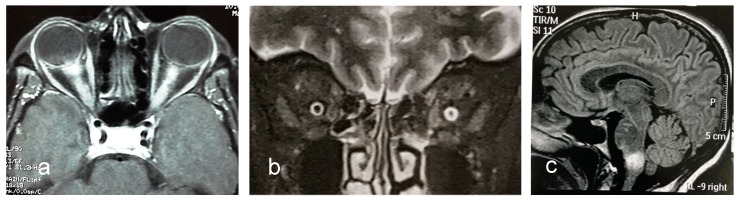
Examples of MRI abnormalities in anti-MOG syndrome. (**a**). Axial T1-weighted MRI reveals longitudinal extensive gadolinium enhancement of both optic nerves. (**b**). Coronal T2-weighted MRI shows hyperintense thickening of perioptic nerve sheath. (**c**). Sagittal T2/FLAIR-weighted image shows large fluffy lesion in the medulla.

Optic neuritis in anti-MOG syndrome exhibits some peculiar features that may distinguish it from optic neuritis in AQP4-IgG NMOSD and MS. Bilateral optic nerve lesions occur more commonly in MOG (and AQP4-IgG) optic neuritis than in MS optic neuritis (Figure 3a). Usually, lesions are longitudinally extensive and tend to locate in the retrobulbar and orbital segments of the optic nerve. Chiasmal involvement is very rare. Perioptic contrast enhancement which may extend to surrounding orbital tissues (Figure 3b) is observed in over one third of patients [132].

Spinal MRI shows in patients with acute myelitis at disease onset LETM lesions in two thirds lesions occur and short lesions (<3 vertebral segments) in one third of patients. Swelling and contrast enhancement of the lesions are frequently observed [131].

Brain MRI is normal in a large majority of anti-MOG NMOSD patients. However, when brain MRI is abnormal, some lesion characteristics may discriminate between anti-MOG NMOSD and MS with high predictive values [96]. Imaging features that are useful to differ between the two conditions are the presence of three lesions or less, and of fluffy brainstem lesions in the pons/or adjacent to fourth ventricle (anti-MOG syndrome) (Figure 3c); and of ovoid lesions adjacent to the body of lateral ventricles, or Dawson’s fingers T1 hypointense lesions (MS). On the other hand, brain MRI does not discriminate anti-MOG-NMOSD from AQP4-IgG NMOSD [96].

### 3.7. Diagnosis

Recently, an international panel of experts [133] formulated the diagnostic criteria for MOG-related disorders in adults. Accordingly, MOG-related disorders should be diagnosed in patients who meet all of the following criteria:

1. Monophasic or relapsing acute ON, myelitis, brainstem encephalitis, or any combination of these symptoms
2. MRI or electrophysiological (visual evoked potentials in patients with isolated ON) findings compatible with CNS demyelination
3. Seropositivity for MOG-IgG as detected by means of a cell-based assay employing full length human MOG as target antigen.

Clinical, laboratory and imaging features that favor the diagnosis of conditions other than MOG-related disorders (“red-flags”) include:

- Chronic progressive course (progressive MS, sarcoidosis and tumors) or acute onset (ischemia);
- Clinical and paraclinical findings suggesting other conditions such as:
  - Tuberculosis, borreliosis, syphilis, Behçet’s disease, subacute combined degeneration of the spinal cord, Leber’s hereditary optic neuropathy, lymphoma, and paraneoplastic disorders;
  - Peripheral demyelination
- Brain MRI abnormalities such as:
  - Lesion adjacent to lateral ventricle associated with inferior temporal lobe lesion, or Dawson’s finger-type lesion;
  - Increasing number of lesions between relapses.
- Serum MOG-IgG at low titers.

It is recommended that patients who test positive for MOG-IgG but in whom a “red flag” is suspected undergo retesting, preferably employing a different CBA [133].

### 3.8. Treatment

Patients with anti-MOG syndrome are usually responsive to steroids, but frequently relapse after prednisone withdrawal or with a rapid taper [134]. More severe attacks or those with suboptimal response to steroid may be treated with plasma exchange or IV immunoglobulin. As relapsing disease is the rule with extended follow-up long-term immunosuppression should follow first-line treatment [132]. Azathioprine, mycophenolate mofetil and rituximab have all been used but studies on their comparative efficacy are still lacking. Multicenter studies are needed to provide physicians with more robust data on the most appropriate way to treat this rare condition.

### 3.9. Conclusions

The understanding of NMOSD has enormously advanced in the last few years. Pathophysiological and clinical studies have cleared up a number of uncertainties and deeply changed the concept of the disease. Previously considered as a variant of MS, characterized by monophasic course and exclusive involvement of the optic nerves and spinal cord, NMOSD is now recognized as an independent disorder, most frequently with relapsing course and a variety of clinical manifestations. The 2015 diagnostic criteria [35] allows for the identification of NMOSD in both patients with AQP4-IgG seropositivity and without the antibody, or who were not tested. High doses of IV steroids and PLEX are the main therapeutic measures during relapses, whereas immunosupressive drugs and rituximab are most useful to prevent new attacks. Monoclonal antibodies and tolerization are emerging and promising therapeutic approaches. Recently, MOG-IgG was identified in patients with relapsing optic neurits, acute myelitis, NMOSD phenotypes, and brainstem encephalitis, in addition to ADEM. Although these patients are treated with IV corticosteroids and immunosupressive agents, data are too scanty to evaluate the real efficacy of these drugs.

As NMOSD and anti-MOG syndromes are rare conditions, international collaborative efforts are necessary to determine their distribution in different regions and populations, their intimate pathophysiological mechanisms and the most efficacious therapeutic approach, in order to improving patients care.

## Figures and Tables

**Table 1:** Incidence and prevalence of NMOSD across the world.

Authors, Year	Country	Number of Cases	Incidence (95% CI) (per Million per Year)	Prevalence (95% CI) (per 100,000)
Rivera et al., 2008 [54]	Mexico	34	0.20 (0.05–0.35)	1
Cabrera-Gómez et al., 2009 [56]	Cuba	58	0.44 (0.3–0.62)	0.43 (0.29–0.61)
Asgari et al., 2011 [55]	Denmark	42	4 (3.0–5.4)	4.41 (3.1–5.7)
Aboul Enein et al., 2011 [57]	Austria	71	0.54 (0.01–0.03)	0.71 (0.17–0.96)
Cossburn et al., 2012 [58]	UK	14	NA	1.96 (1.22–2.97)
Houzen et al., 2012 [59]	Japan	3	0.8 (0.3–1.6)	0.72 (0.31–1.42)
Jacob et al., 2013 [60]	UK	13	0.8 (0.3–1.6)	0.72 (0.31–1.42)
Etemadifar et al., 2014 [61]	Iran	95	NA	1.95 (1.62–2.23)
Pandit et al., 2014 [62]	India	11	NA	2.6
Kashipazha et al., 2015 [63]	Iran	51	NA	0.8 (0.54–1.06)
Flanagan et al., 2016 [53]	USA	6	0.7 (0.0–2.1)	3.9 (0.8–7.1)
	Martinique	39	7.3 (4.1–10.1)	10.0 (6.8–13.2)
van Pelt et al., 2016 [64]	Netherlands		1.2	NA
Houzen et al., 2017 [65]	Japan	14	NA	4.1 (2.2–6.9)
Hor et al., 2017 [66]	Malaysia	14	NA	1.99 (1.09–3.35)
Bukhari et al., 2017 [67]	ANZ	81	0.37 (0.36–0.38)	0.7 (0.66–0.74)
Sepulveda et al., 2017 [68]	Spain	74	0.63 (0.45–0.8)	0.89 (0.87–0.91)
Holroyd et al., 2018 [69]	United Arab Emirates	10	0.59	0.34

NMOSD: neuromyelitis optica spectrum disorders; NA: not available; ANZ: Australia and New Zealand.

**Table 2:** Clinical manifestations of NMOSD according to anatomic involvement *.

Site	Symtoms
Optic nerve/chiasm	Eye pain or headache
	Blurred vision
	Disturbance of color vision
	Amaurosis
	Optic disc edema
	Optic atrophy
	Scotomas and other visual field defects
Spinal cord	Limb weakness
	Lower limb spasticity
	Gait abnormalities
	Sensory disturbances
	Radicular pain
	Pruritus
	Painful tonic spasms
	Trunk and limb ataxia
	Sphincter disturbances
	Respiratory weakness
	Lhermitte phenomenon
Brainstem	Motor and sensory disturbances
	Incoercible nausea, vomiting and hiccups
	Intractable cough
	Weight loss
	Anorexia
	Diplopia/ocular movement disorders
	Facial dysesthesia and trigeminal neuralgia
	Dysgeusia
	Facial paralysis
	Hearing loss, tinnitus
	Vertigo
	Dysarthria/dysphagia
Diencephalon	Narcolepsy
	Hypophyseal abnormalities
	Antidiuretic hormone syndrome
	Pre-syncopal symptoms
	Disturbances of body temperature
	Anhydrosis/excessive sweating
	Hyperphagia
Cerebrum	Posterior reversible encephalopathy syndrome (PRES)
	Mental confusion
	Seizures
	Aphasia
	Apraxia
	Cognitive dysfunction
	Psychiatric symptoms

* Modified from Lana-Peixoto and Callegaro, 2012 [34]. NMOSD: neuromyelitis optica spectrum disorders.

**Table 3:** International consensus diagnostic criteria for NMOSD *.

**1. Diagnostic criteria for NMOSD with AQP4-IgG**			
	1. At least one core clinical characteristic		
	2. Exclusion of alternative diagnoses		
**2. Diagnostic criteria for NMOSD without AQP4-IgG or NMOSD with unknown AQP4-IgG status**			
	1. At least two core clinical characteristics meeting all of the following requirements:		
	a. At least one core clinical characteristic must be optic neuritis, acute myelitis with LETM, or area postrema syndrome		
	b. Dissemination in space (two or more different core clinical characteristics)		
	c. Core clinical syndromes must be associated with respective MRI findings:		
		i. Optic neuritis:	
			1. Brain MRI is normal or with nonspecific lesions; OR
			2. Optic nerve lesion extending over ½ of the optic nerve length; or chiasmal lesion
		ii. Acute myelitis: MRI with lesion or spinal atrophy extending over ≥3 contiguous segments	
		iii. Area postrema syndrome: MRI with dorsal medulla/area postrema lesions	
		iv. Acute brainstem syndrome: MRI with periependymal brainstem lesions	
		v. Narcolepsy or acute diencephalic clinical syndrome: MRI with NMOSD-typical diencephalic lesions	
	2. Exclusion of alternative diagnoses		

* Modified from Wingerchuk et al., 2015 [35]. NMOSD: neuromyelitis optica spectrum disorders; AQP4: aquaporin-4; IgG: immunoglobulin G; LETM: longitudinally extensive transverse myelitis lesions.

**Table 4:** Distinctive characteristics between MS and NMOSD.

**Distinctive characteristics of MS**	
Progressive course	
Partial transverse myelitis	
Brain MRI features	
	Perpendicular periventricular lesions (Dawson fingers)
	Periventricular lesions in the inferior temporal lobe
	Juxtacortical lesions involving subcortical U-fibers
	Cortical lesions
	More severe brain atrophy
Spinal cord MRI features	
	Lesions <3 complete vertebral segments
	Lesions located predominantly in the peripheral cord
	Diffuse, indistinct signal change on T2-weighted sequences
Cerebrospinal fluid analysis	
	Presence of oligoclonal bands
Optic coherence tomography features	
	Predominant atrophy of temporal RNFL
**Distinctive characteristics of NMOSD**	
Complete transverse myelitis	
Brain MRI features	
	Multiple patchy enhancement with blurred margin in adjacent regions (cloud-like enhancement)
	Large and edematous callosal lesions
	Large and confluent white matter lesions (as in PRES)
	Predominantly posterior brainstem lesions (around the fourth ventricle lesions and periaqueductal lesions)
	Hypothalamic lesions
	Extensive optic nerve lesions and chiasmal lesions
Spinal cord MRI features	
	Longitudinally extensive transverse myelitis lesions (≥3 contiguous segments)
	Longitudinally extensive spinal cord atrophy (≥3 contiguous segments)
	Centrally-located or holomedullary spinal cord lesions
Cerebrospinal fluid analysis	
	Moderate or marked pleocytosis
	Presence of neutrophils and eosinophils
Optic coherence tomography features	
	Predominant atrophy of superior and inferior RNFL

MS: multiple sclerosis; NMOSD: neuromyelitis optica spectrum disorders; PRES: posterior reversible encephalopathy syndrome; RNFL: retinal nerve fiber layer.

**Table 5:** Differential diagnosis of NMOSD.

Multiple Sclerosis	
Acute disseminated encephalomyelitis	
MOG-related disorders	
Sarcoidosis	
Lymphoma	
Paraneoplastic disease	
Central nervous system infections	
Syphilis	
	Tuberculosis
	Human T-lymphotropic virus-I (HTLV-I) infection
	Herpes virus infection
	Dengue-virus infection
	Lyme disease
	Schistosomiasis
Sjogren syndrome	
Systemic lupus erythematosus	
Neuro-Behçet’s disease	
Spinal dural arteriovenous fistula	

NMOSD: neuromyelitis optica spectrum disorders.

**Table 6:** Drugs used in relapse prevention in neuromyelitis optica spectrum disorders.

Drugs	Route	Regimen	Comments
Prednisone	Oral	≥30 mg/d	Keep until until azathioprine or mycophenolate fully effective, then taper over six months
Azathioprine	Oral	2-3 mg/kg/d in 2 doses	First line treatment; latency four to six months; target dose guided by ALC and MCV; monitor liver function
Mycophenolate mofetil	Oral	1500–3000 mg/d in 2 doses	Target dose guided by ALC and blood concentration (1–2 μg/mL)
Rituximab	IV	1000 mg given twice, 14 d apart.Repeat every 6 mo or based on reemergence of CD19 B cells	First-line therapy; CD19 B cells as a marker
Methotrexate	Oral	15–25.0 mg weekly	Supplement with folic acid 1 mg/d, monitor liver function
Ciclosporin A	Oral	2–5 mg/kg/day in 2 doses	Nephrotoxic, target dose guided by blood concentration (70–100 ng/mL)
Tacrolimus	Oral	1–6 mg/day in 2 doses	Nephrotoxic, target dose guided by blood concentration (5–10 ng/mL)
Mitoxantrone	IV	12 mg/m2 every 1–3 months	Cardiac monitoring (LVEF), target dose guided by leukocyte count; total cumulative dose 100 mg/m2
Tocilizumab	IV	8 mg/kg every 4 weeks	8 mg/kg every four weeks; monitoring for infections; CRP no reliable biomarker for infection

ALC = absolute lymphocyte count; MCV = mean corpuscular volume; IV = intravenously; LVEF = left ventricular ejection fraction; CRP = C-reactive protein.

## References

[1] Kleiter I., Hellwig K., Berthele A., Kumpfel T., Linker R.A., Hartung H.P., Paul F., Aktas O. (2012). Failure of natalizumab to prevent relapses in neuromyelitis optica. Arch. Neurol..

[2] Min J.H., Kim B.J., Lee K.H. (2012). Development of extensive brain lesions following fingolimod (fty720) treatment in a patient with neuromyelitis optica spectrum disorder. Mult. Scler..

[3] Trebst C., Jarius S., Berthele A., Paul F., Schippling S., Wildemann B., Borisow N., Kleiter I., Aktas O., Kumpfel T. (2014). Update on the diagnosis and treatment of neuromyelitis optica: Recommendations of the neuromyelitis optica study group (nemos). J. Neurol..

[4] van Pelt E.D., Wong Y.Y., Ketelslegers I.A., Hamann D., Hintzen R.Q. (2016). Neuromyelitis optica spectrum disorders: Comparison of clinical and magnetic resonance imaging characteristics of aqp4-igg versus mog-igg seropositive cases in the netherlands. Eur. J. Neurol..

[5] Narayan R., Simpson A., Fritsche K., Salama S., Pardo S., Mealy M., Paul F., Levy M. (2018). Mog antibody disease: A review of mog antibody seropositive neuromyelitis optica spectrum disorder. Mult. Scler. Relat. Disord..

[6] Jarius S., Metz I., Konig F.B., Ruprecht K., Reindl M., Paul F., Bruck W., Wildemann B. (2016). Screening for mog-igg and 27 other anti-glial and anti-neuronal autoantibodies in ‘pattern ii multiple sclerosis’ and brain biopsy findings in a mog-igg-positive case. Mult. Scler..

[7] Spadaro M., Gerdes L.A., Mayer M.C., Ertl-Wagner B., Laurent S., Krumbholz M., Breithaupt C., Hogen T., Straube A., Giese A. (2015). Histopathology and clinical course of mog-antibody-associated encephalomyelitis. Ann. Clin. Transl. Neurol..

[8] Misu T., Hoftberger R., Fujihara K., Wimmer I., Takai Y., Nishiyama S., Nakashima I., Konno H., Bradl M., Garzuly F. (2013). Presence of six different lesion types suggests diverse mechanisms of tissue injury in neuromyelitis optica. Acta Neuropathol..

[9] Reindl M., Rostasy K. (2015). Mog antibody-associated diseases. Neurol. Neuroimmunol. Neuroinflamm..

[10] Di Pauli F., Hoftberger R., Reindl M., Beer R., Rhomberg P., Schanda K., Sato D., Fujihara K., Lassmann H., Schmutzhard E. (2015). Fulminant demyelinating encephalomyelitis: Insights from antibody studies and neuropathology. Neurol. Neuroimmunol. Neuroinflamm..

[11] Ramanathan S., Dale R.C., Brilot F. (2016). Anti-mog antibody: The history, clinical phenotype, and pathogenicity of a serum biomarker for demyelination. Autoimmun. Rev..

[12] Jarius S., Probst C., Borowski K., Franciotta D., Wildemann B., Stoecker W., Wandinger K.P. (2010). Standardized method for the detection of antibodies to aquaporin-4 based on a highly sensitive immunofluorescence assay employing recombinant target antigen. J. Neurol. Sci..

[13] Waters P.J., McKeon A., Leite M.I., Rajasekharan S., Lennon V.A., Villalobos A., Palace J., Mandrekar J.N., Vincent A., Bar-Or A. (2012). Serologic diagnosis of nmo: A multicenter comparison of aquaporin-4-igg assays. Neurology.

[14] Waters P.J., Pittock S.J., Bennett J.L., Jarius S., Weinshenker B.G., Wingerchuk D.M. (2014). Evaluation of aquaporin-4 antibody assays. Clin. Exp. Neuroimmunol..

[15] Jarius S., Ruprecht K., Kleiter I., Borisow N., Asgari N., Pitarokoili K., Pache F., Stich O., Beume L.A., Hummert M.W. (2016). Mog-igg in nmo and related disorders: A multicenter study of 50 patients. Part 1: Frequency, syndrome specificity, influence of disease activity, long-term course, association with aqp4-igg, and origin. J. Neuroinflamm..

[16] Ishikawa N., Tajima G., Hyodo S., Takahashi Y., Kobayashi M. (2007). Detection of autoantibodies against nmda-type glutamate receptor in a patient with recurrent optic neuritis and transient cerebral lesions. Neuropediatrics.

[17] Kruer M.C., Koch T.K., Bourdette D.N., Chabas D., Waubant E., Mueller S., Moscarello M.A., Dalmau J., Woltjer R.L., Adamus G. (2010). Nmda receptor encephalitis mimicking seronegative neuromyelitis optica. Neurology.

[18] Mader S., Gredler V., Schanda K., Rostasy K., Dujmovic I., Pfaller K., Lutterotti A., Jarius S., Di Pauli F., Kuenz B. (2011). Complement activating antibodies to myelin oligodendrocyte glycoprotein in neuromyelitis optica and related disorders. J. Neuroinflamm..

[19] Jarius S., Wandinger K.P., Borowski K., Stoecker W., Wildemann B. (2012). Antibodies to cv2/crmp5 in neuromyelitis optica-like disease: Case report and review of the literature. Clin. Neurol. Neurosurg..

[20] Devic E. (1894). Myelite subaigue compliquee de nevrite optique. Bull. Med..

[21] Gault F. (1894). De la neuromyélite optique aiguë. Ph.D. Thesis.

[22] Acchiote P. (1907). Sur un cas de neuromyélite subaiguë ou maladie de devic. Rev. Neurol..

[23] Jarius S., Wildemann B. (2012). The case of the marquis de causan (1804): An early account of visual loss associated with spinal cord inflammation. J. Neurol..

[24] Jarius S., Wildemann B. (2012). ‘Noteomielite’ accompanied by acute amaurosis (1844). An early case of neuromyelitis optica. J. Neurol. Sci..

[25] Jarius S., Wildemann B. (2012). An early british case of neuromyelitis optica (1850). BMJ Clin. Res. Ed..

[26] Jarius S., Wildemann B. (2011). An early case of neuromyelitis optica: On a forgotten report by jacob lockhart clarke, frs. Mult. Scler..

[27] Allbutt T.C. (1870). On the ophthalmoscopic signs of spinal disease. Lancet.

[28] Erb W. (1880). Ueber das zusammenvorkommen von neuritis optica und myelitis subacuta. Eur. Arch. Psychiatry Clin. Neurosci..

[29] Seguin E.C. (1880). Art. I.—On the coincidence of optic neuritis and subacute transverse myelitis. J. Nerv. Ment. Dis..

[30] Marques A. (1943). Da neuromielite ótica: Contribuição clínica e etiológica. Hospital.

[31] Lennon V.A., Wingerchuk D.M., Kryzer T.J., Pittock S.J., Lucchinetti C.F., Fujihara K., Nakashima I., Weinshenker B.G. (2004). A serum autoantibody marker of neuromyelitis optica: Distinction from multiple sclerosis. Lancet.

[32] Lennon V.A., Kryzer T.J., Pittock S.J., Verkman A.S., Hinson S.R. (2005). Igg marker of optic-spinal multiple sclerosis binds to the aquaporin-4 water channel. J. Exp. Med..

[33] Wingerchuk D.M., Lennon V.A., Lucchinetti C.F., Pittock S.J., Weinshenker B.G. (2007). The spectrum of neuromyelitis optica. Lancet Neurol..

[34] Lana-Peixoto M.A., Callegaro D. (2012). The expanded spectrum of neuromyelitis optica: Evidences for a new definition. Arq. Neuro-Psiquiatr..

[35] Wingerchuk D.M., Banwell B., Bennett J.L., Cabre P., Carroll W., Chitnis T., de Seze J., Fujihara K., Greenberg B., Jacob A. (2015). International consensus diagnostic criteria for neuromyelitis optica spectrum disorders. Neurology.

[36] Jin B.J., Rossi A., Verkman A.S. (2011). Model of aquaporin-4 supramolecular assembly in orthogonal arrays based on heterotetrameric association of m1-m23 isoforms. Biophys. J..

[37] Papadopoulos M.C., Verkman A.S. (2012). Aquaporin 4 and neuromyelitis optica. Lancet Neurol..

[38] Verkman A.S., Anderson M.O., Papadopoulos M.C. (2014). Aquaporins: Important but elusive drug targets. Nat. Rev. Drug Discov..

[39] Saadoun S., Waters P., Bell B.A., Vincent A., Verkman A.S., Papadopoulos M.C. (2010). Intra-cerebral injection of neuromyelitis optica immunoglobulin g and human complement produces neuromyelitis optica lesions in mice. Brain J. Neurol..

[40] Lucchinetti C.F., Mandler R.N., McGavern D., Bruck W., Gleich G., Ransohoff R.M., Trebst C., Weinshenker B., Wingerchuk D., Parisi J.E. (2002). A role for humoral mechanisms in the pathogenesis of devic’s neuromyelitis optica. Brain J. Neurol..

[41] Jarius S., Franciotta D., Paul F., Ruprecht K., Bergamaschi R., Rommer P.S., Reuss R., Probst C., Kristoferitsch W., Wandinger K.P. (2010). Cerebrospinal fluid antibodies to aquaporin-4 in neuromyelitis optica and related disorders: Frequency, origin, and diagnostic relevance. J. Neuroinflamm..

[42] Chihara N., Aranami T., Sato W., Miyazaki Y., Miyake S., Okamoto T., Ogawa M., Toda T., Yamamura T. (2011). Interleukin 6 signaling promotes anti-aquaporin 4 autoantibody production from plasmablasts in neuromyelitis optica. Proc. Natl. Acad. Sci. USA.

[43] Shimizu F., Schaller K.L., Owens G.P., Cotleur A.C., Kellner D., Takeshita Y., Obermeier B., Kryzer T.J., Sano Y., Kanda T. (2017). Glucose-regulated protein 78 autoantibody associates with blood-brain barrier disruption in neuromyelitis optica. Sci. Transl. Med..

[44] Shimizu F., Nishihara H., Kanda T. (2018). Blood-brain barrier dysfunction in immuno-mediated neurological diseases. Immunol. Med..

[45] Jarius S., Wildemann B., Paul F. (2014). Neuromyelitis optica: Clinical features, immunopathogenesis and treatment. Clin. Exp. Immunol..

[46] Weinshenker B.G., Wingerchuk D.M. (2017). Neuromyelitis spectrum disorders. Mayo Clin. Proc..

[47] Hinson S.R., Pittock S.J., Lucchinetti C.F., Roemer S.F., Fryer J.P., Kryzer T.J., Lennon V.A. (2007). Pathogenic potential of igg binding to water channel extracellular domain in neuromyelitis optica. Neurology.

[48] Bennett J.L., Lam C., Kalluri S.R., Saikali P., Bautista K., Dupree C., Glogowska M., Case D., Antel J.P., Owens G.P. (2009). Intrathecal pathogenic anti-aquaporin-4 antibodies in early neuromyelitis optica. Ann. Neurol..

[49] Bradl M., Misu T., Takahashi T., Watanabe M., Mader S., Reindl M., Adzemovic M., Bauer J., Berger T., Fujihara K. (2009). Neuromyelitis optica: Pathogenicity of patient immunoglobulin in vivo. Ann. Neurol..

[50] Kinoshita M., Nakatsuji Y., Kimura T., Moriya M., Takata K., Okuno T., Kumanogoh A., Kajiyama K., Yoshikawa H., Sakoda S. (2009). Neuromyelitis optica: Passive transfer to rats by human immunoglobulin. Biochem. Biophys. Res. Commun..

[51] Kinoshita M., Nakatsuji Y., Kimura T., Moriya M., Takata K., Okuno T., Kumanogoh A., Kajiyama K., Yoshikawa H., Sakoda S. (2010). Anti-aquaporin-4 antibody induces astrocytic cytotoxicity in the absence of cns antigen-specific t cells. Biochem. Biophys. Res. Commun..

[52] Etemadifar M., Nasr Z., Khalili B., Taherioun M., Vosoughi R. (2015). Epidemiology of neuromyelitis optica in the world: A systematic review and meta-analysis. Mult. Scler. Int..

[53] Flanagan E.P., Cabre P., Weinshenker B.G., Sauver J.S., Jacobson D.J., Majed M., Lennon V.A., Lucchinetti C.F., McKeon A., Matiello M. (2016). Epidemiology of aquaporin-4 autoimmunity and neuromyelitis optica spectrum. Ann. Neurol..

[54] Rivera J.F., Kurtzke J.F., Booth V.A., Corona T. (2008). Characteristics of devic’s disease (neuromyelitis optica) in mexico. J. Neurol..

[55] Asgari N., Lillevang S.T., Skejoe H.P., Falah M., Stenager E., Kyvik K.O. (2011). A population-based study of neuromyelitis optica in caucasians. Neurology.

[56] Cabrera-Gomez J.A., Kurtzke J.F., Gonzalez-Quevedo A., Lara-Rodriguez R. (2009). An epidemiological study of neuromyelitis optica in cuba. J. Neurol..

[57] Aboul-Enein F., Seifert-Held T., Mader S., Kuenz B., Lutterotti A., Rauschka H., Rommer P., Leutmezer F., Vass K., Flamm-Horak A. (2013). Neuromyelitis optica in austria in 2011: To bridge the gap between neuroepidemiological research and practice in a study population of 8.4 million people. PLoS ONE.

[58] Cossburn M., Tackley G., Baker K., Ingram G., Burtonwood M., Malik G., Pickersgill T., te Water Naude J., Robertson N. (2012). The prevalence of neuromyelitis optica in south east wales. Eur. J. Neurol..

[59] Houzen H., Niino M., Hirotani M., Fukazawa T., Kikuchi S., Tanaka K., Sasaki H. (2012). Increased prevalence, incidence, and female predominance of multiple sclerosis in northern japan. J. Neurol. Sci..

[60] Jacob A., Panicker J., Lythgoe D., Elsone L., Mutch K., Wilson M., Das K., Boggild M. (2013). The epidemiology of neuromyelitis optica amongst adults in the merseyside county of united kingdom. J. Neurol..

[61] Etemadifar M., Dashti M., Vosoughi R., Abtahi S.H., Ramagopalan S.V., Nasr Z. (2014). An epidemiological study of neuromyelitis optica in isfahan. Mult. Scler..

[62] Pandit L., Kundapur R. (2014). Prevalence and patterns of demyelinating central nervous system disorders in urban mangalore, south india. Mult. Scler..

[63] Kashipazha D., Mohammadianinejad S.E., Majdinasab N., Azizi M., Jafari M. (2015). A descriptive study of prevalence, clinical features and other findings of neuromyelitis optica and neuromyelitis optica spectrum disorder in khuzestan province, iran. Iran. J. Neurol..

[64] Danielle van Pelt E., Wong Y.Y.M., Ketelslegers I.A., Siepman D.A., Hamann D., Hintzen R.Q. (2016). Incidence of aqp4-igg seropositive neuromyelitis optica spectrum disorders in the netherlands: About one in a million. Mult. Scler. J. Exp. Transl. Clin..

[65] Houzen H., Kondo K., Niino M., Horiuchi K., Takahashi T., Nakashima I., Tanaka K. (2017). Prevalence and clinical features of neuromyelitis optica spectrum disorders in northern japan. Neurology.

[66] Hor J.Y., Lim T.T., Chia Y.K., Ching Y.M., Cheah C.F., Tan K., Chow H.B., Arip M., Eow G.B., Easaw P.E.S. (2018). Prevalence of neuromyelitis optica spectrum disorder in the multi-ethnic penang island, malaysia, and a review of worldwide prevalence. Mult. Scler. Relat. Disord..

[67] Bukhari W., Prain K.M., Waters P., Woodhall M., O’Gorman C.M., Clarke L., Silvestrini R.A., Bundell C.S., Abernethy D., Bhuta S. (2017). Incidence and prevalence of nmosd in australia and new zealand. J. Neurol. Neurosurg. Psychiatry.

[68] Sepulveda M., Aldea M., Escudero D., Llufriu S., Arrambide G., Otero-Romero S., Sastre-Garriga J., Romero-Pinel L., Martinez-Yelamos S., Sola-Valls N. (2017). Epidemiology of nmosd in catalonia: Influence of the new 2015 criteria in incidence and prevalence estimates. Mult. Scler..

[69] Holroyd K.B., Aziz F., Szolics M., Alsaadi T., Levy M., Schiess N. (2018). Prevalence and characteristics of transverse myelitis and neuromyelitis optica spectrum disorders in the united arab emirates: A multicenter, retrospective study. Clin. Exp. Neuroimmunol..

[70] Mori M., Kuwabara S., Paul F. (2018). Worldwide prevalence of neuromyelitis optica spectrum disorders. J. Neurol. Neurosurg. Psychiatry.

[71] Kim S.H., Mealy M.A., Levy M., Schmidt F., Ruprecht K., Paul F., Ringelstein M., Aktas O., Hartung H.P., Asgari N. (2018). Racial differences in neuromyelitis optica spectrum disorder. Neurology.

[72] Kitley J., Leite M.I., Nakashima I., Waters P., McNeillis B., Brown R., Takai Y., Takahashi T., Misu T., Elsone L. (2012). Prognostic factors and disease course in aquaporin-4 antibody-positive patients with neuromyelitis optica spectrum disorder from the united kingdom and japan. Brain J. Neurol..

[73] Sepulveda M., Armangue T., Sola-Valls N., Arrambide G., Meca-Lallana J.E., Oreja-Guevara C., Mendibe M., Alvarez de Arcaya A., Aladro Y., Casanova B. (2016). Neuromyelitis optica spectrum disorders: Comparison according to the phenotype and serostatus. Neurol. Neuroimmunol. Neuroinflamm..

[74] McKeon A., Lennon V.A., Lotze T., Tenenbaum S., Ness J.M., Rensel M., Kuntz N.L., Fryer J.P., Homburger H., Hunter J. (2008). Cns aquaporin-4 autoimmunity in children. Neurology.

[75] Matiello M., Kim H.J., Kim W., Brum D.G., Barreira A.A., Kingsbury D.J., Plant G.T., Adoni T., Weinshenker B.G. (2010). Familial neuromyelitis optica. Neurology.

[76] Matsushita T., Matsuoka T., Isobe N., Kawano Y., Minohara M., Shi N., Nishimura Y., Ochi H., Kira J. (2009). Association of the hla-dpb1*0501 allele with anti-aquaporin-4 antibody positivity in japanese patients with idiopathic central nervous system demyelinating disorders. Tissue Antigens.

[77] Wang H., Dai Y., Qiu W., Zhong X., Wu A., Wang Y., Lu Z., Bao J., Hu X. (2011). Hla-dpb1 0501 is associated with susceptibility to anti-aquaporin-4 antibodies positive neuromyelitis optica in southern han chinese. J. Neuroimmunol..

[78] Blanco Y., Ercilla-Gonzalez G., Llufriu S., Casanova-Estruch B., Magraner M.J., Ramio-Torrenta L., Mendibe-Bilbao M.M., Ucles-Sanchez A.J., Casado-Chocan J.L., Lopez de Munain A. (2011). hla-drb1 typing in caucasians patients with neuromyelitis optica. Rev. De Neurol..

[79] Pandit L., Malli C., D’Cunha A., Mustafa S. (2015). Human leukocyte antigen association with neuromyelitis optica in a south indian population. Mult. Scler..

[80] Deschamps R., Paturel L., Jeannin S., Chausson N., Olindo S., Bera O., Bellance R., Smadja D., Cesaire D., Cabre P. (2011). Different hla class ii (drb1 and dqb1) alleles determine either susceptibility or resistance to nmo and multiple sclerosis among the french afro-caribbean population. Mult. Scler..

[81] Zephir H., Fajardy I., Outteryck O., Blanc F., Roger N., Fleury M., Rudolf G., Marignier R., Vukusic S., Confavreux C. (2009). Is neuromyelitis optica associated with human leukocyte antigen?. Mult. Scler..

[82] Popescu B.F., Lennon V.A., Parisi J.E., Howe C.L., Weigand S.D., Cabrera-Gomez J.A., Newell K., Mandler R.N., Pittock S.J., Weinshenker B.G. (2011). Neuromyelitis optica unique area postrema lesions: Nausea, vomiting, and pathogenic implications. Neurology.

[83] Dubey D., Pittock S.J., Krecke K.N., Flanagan E.P. (2017). Association of extension of cervical cord lesion and area postrema syndrome with neuromyelitis optica spectrum disorder. JAMA Neurol..

[84] Kremer L., Mealy M., Jacob A., Nakashima I., Cabre P., Bigi S., Paul F., Jarius S., Aktas O., Elsone L. (2014). Brainstem manifestations in neuromyelitis optica: A multicenter study of 258 patients. Mult. Scler..

[85] Jarius S., Wildemann B. (2013). Aquaporin-4 antibodies (nmo-igg) as a serological marker of neuromyelitis optica: A critical review of the literature. Brain Pathol..

[86] Marignier R., Bernard-Valnet R., Giraudon P., Collongues N., Papeix C., Zephir H., Cavillon G., Rogemond V., Casey R., Frangoulis B. (2013). Aquaporin-4 antibody-negative neuromyelitis optica: Distinct assay sensitivity-dependent entity. Neurology.

[87] Pittock S.J., Lennon V.A., Bakshi N., Shen L., McKeon A., Quach H., Briggs F.B., Bernstein A.L., Schaefer C.A., Barcellos L.F. (2014). Seroprevalence of aquaporin-4-igg in a northern california population representative cohort of multiple sclerosis. JAMA Neurol..

[88] Waters P., Reindl M., Saiz A., Schanda K., Tuller F., Kral V., Nytrova P., Sobek O., Nielsen H.H., Barington T. (2016). Multicentre comparison of a diagnostic assay: Aquaporin-4 antibodies in neuromyelitis optica. J. Neurol. Neurosurg. Psychiatry.

[89] Andersson M., Alvarez-Cermeno J., Bernardi G., Cogato I., Fredman P., Frederiksen J., Fredrikson S., Gallo P., Grimaldi L.M., Gronning M. (1994). Cerebrospinal fluid in the diagnosis of multiple sclerosis: A consensus report. J. Neurol. Neurosurg. Psychiatry.

[90] Jarius S., Ruprecht K., Wildemann B., Kuempfel T., Ringelstein M., Geis C., Kleiter I., Kleinschnitz C., Berthele A., Brettschneider J. (2012). Contrasting disease patterns in seropositive and seronegative neuromyelitis optica: A multicentre study of 175 patients. J. Neuroinflamm..

[91] Kim S.M., Kim S.J., Lee H.J., Kuroda H., Palace J., Fujihara K. (2017). Differential diagnosis of neuromyelitis optica spectrum disorders. Ther. Adv. Neurol. Disord..

[92] Flanagan E.P., Weinshenker B.G., Krecke K.N., Lennon V.A., Lucchinetti C.F., McKeon A., Wingerchuk D.M., Shuster E.A., Jiao Y., Horta E.S. (2015). Short myelitis lesions in aquaporin-4-igg-positive neuromyelitis optica spectrum disorders. JAMA Neurol..

[93] Pittock S.J., Weinshenker B.G., Lucchinetti C.F., Wingerchuk D.M., Corboy J.R., Lennon V.A. (2006). Neuromyelitis optica brain lesions localized at sites of high aquaporin 4 expression. Arch. Neurol..

[94] Matthews L., Marasco R., Jenkinson M., Kuker W., Luppe S., Leite M.I., Giorgio A., De Stefano N., Robertson N., Johansen-Berg H. (2013). Distinction of seropositive nmo spectrum disorder and ms brain lesion distribution. Neurology.

[95] Kim H.J., Paul F., Lana-Peixoto M.A., Tenembaum S., Asgari N., Palace J., Klawiter E.C., Sato D.K., de Seze J., Wuerfel J. (2015). Mri characteristics of neuromyelitis optica spectrum disorder: An international update. Neurology.

[96] Jurynczyk M., Tackley G., Kong Y., Geraldes R., Matthews L., Woodhall M., Waters P., Kuker W., Craner M., Weir A. (2017). Brain lesion distribution criteria distinguish ms from aqp4-antibody nmosd and mog-antibody disease. J. Neurol. Neurosurg. Psychiatry.

[97] Kira J.I. (2017). Unexpected exacerbations following initiation of disease-modifying drugs in neuromyelitis optica spectrum disorder: Which factor is responsible, anti-aquaporin 4 antibodies, b cells, th1 cells, th2 cells, th17 cells, or others?. Mult. Scler..

[98] Kleiter I., Gahlen A., Borisow N., Fischer K., Wernecke K.D., Wegner B., Hellwig K., Pache F., Ruprecht K., Havla J. (2016). Neuromyelitis optica: Evaluation of 871 attacks and 1,153 treatment courses. Ann. Neurol..

[99] Weinshenker B.G. (2016). What is the optimal sequence of rescue treatments for attacks of neuromyelitis optica spectrum disorder?. Ann. Neurol..

[100] Wingerchuk D.M., Weinshenker B.G. (2008). Neuromyelitis optica. Curr. Treat. Options Neurol..

[101] Kim S.H., Hyun J.W., Kim H.J. (2018). Individualized b cell-targeting therapy for neuromyelitis optica spectrum disorder. Neurochem. Int..

[102] Paul F., Murphy O., Pardo S., Levy M. (2018). Investigational drugs in development to prevent neuromyelitis optica relapses. Expert Opin. Investig. Drugs.

[103] Kelly R.J., Hochsmann B., Szer J., Kulasekararaj A., de Guibert S., Roth A., Weitz I.C., Armstrong E., Risitano A.M., Patriquin C.J. (2015). Eculizumab in pregnant patients with paroxysmal nocturnal hemoglobinuria. N. Engl. J. Med..

[104] McNamara L.A., Topaz N., Wang X., Hariri S., Fox L., MacNeil J.R. (2017). High risk for invasive meningococcal disease among patients receiving eculizumab (soliris) despite receipt of meningococcal vaccine. Am. J. Transplant. Off. J. Am. Soc. Transplant. Am. Soc. Transpl. Surg..

[105] Traboulsee A., Greenberg B., Bennett J.L., Szczechowiski L., Fox E., Shkrobot S., Yamamura T., Terada Y., Kawata Y., Melia A. A double-blind placebo-controlled study of satralizumab (sa 237), a recycling anti-il-6 receptor monoclonal antibody, as monotherapy for patients witn neuromyelitis optica spectrum disorder (nmosd). Proceedings of the ECTRIMS.

[106] Steinman L., Bar-Or A., Behne J.M., Benitez-Ribas D., Chin P.S., Clare-Salzler M., Healey D., Kim J.I., Kranz D.M., Lutterotti A. (2016). Restoring immune tolerance in neuromyelitis optica: Part i. Neurol. Neuroimmunol. Neuroinflamm..

[107] Brunner C., Lassmann H., Waehneldt T.V., Matthieu J.M., Linington C. (1989). Differential ultrastructural localization of myelin basic protein, myelin/oligodendroglial glycoprotein, and 2’,3’-cyclic nucleotide 3’-phosphodiesterase in the cns of adult rats. J. Neurochem..

[108] Pham-Dinh D., Mattei M.G., Nussbaum J.L., Roussel G., Pontarotti P., Roeckel N., Mather I.H., Artzt K., Lindahl K.F., Dautigny A. (1993). Myelin/oligodendrocyte glycoprotein is a member of a subset of the immunoglobulin superfamily encoded within the major histocompatibility complex. Proc. Natl. Acad. Sci. USA.

[109] Gardinier M.V., Amiguet P., Linington C., Matthieu J.M. (1992). Myelin/oligodendrocyte glycoprotein is a unique member of the immunoglobulin superfamily. J. Neurosci. Res..

[110] Berger T., Rubner P., Schautzer F., Egg R., Ulmer H., Mayringer I., Dilitz E., Deisenhammer F., Reindl M. (2003). Antimyelin antibodies as a predictor of clinically definite multiple sclerosis after a first demyelinating event. N. Engl. J. Med..

[111] Reindl M., Linington C., Brehm U., Egg R., Dilitz E., Deisenhammer F., Poewe W., Berger T. (1999). Antibodies against the myelin oligodendrocyte glycoprotein and the myelin basic protein in multiple sclerosis and other neurological diseases: A comparative study. Brain J. Neurol..

[112] Karni A., Bakimer-Kleiner R., Abramsky O., Ben-Nun A. (1999). Elevated levels of antibody to myelin oligodendrocyte glycoprotein is not specific for patients with multiple sclerosis. Arch. Neurol..

[113] Markovic M., Trajkovic V., Drulovic J., Mesaros S., Stojsavljevic N., Dujmovic I., Mostarica Stojkovic M. (2003). Antibodies against myelin oligodendrocyte glycoprotein in the cerebrospinal fluid of multiple sclerosis patients. J. Neurol. Sci..

[114] Gaertner S., de Graaf K.L., Greve B., Weissert R. (2004). Antibodies against glycosylated native mog are elevated in patients with multiple sclerosis. Neurology.

[115] Lindert R.B., Haase C.G., Brehm U., Linington C., Wekerle H., Hohlfeld R. (1999). Multiple sclerosis: B- and t-cell responses to the extracellular domain of the myelin oligodendrocyte glycoprotein. Brain J. Neurol..

[116] Egg R., Reindl M., Deisenhammer F., Linington C., Berger T. (2001). Anti-mog and anti-mbp antibody subclasses in multiple sclerosis. Mult. Scler..

[117] Kuhle J., Lindberg R.L., Regeniter A., Mehling M., Hoffmann F., Reindl M., Berger T., Radue E.W., Leppert D., Kappos L. (2007). Antimyelin antibodies in clinically isolated syndromes correlate with inflammation in mri and csf. J. Neurol..

[118] Brilot F., Dale R.C., Selter R.C., Grummel V., Kalluri S.R., Aslam M., Busch V., Zhou D., Cepok S., Hemmer B. (2009). Antibodies to native myelin oligodendrocyte glycoprotein in children with inflammatory demyelinating central nervous system disease. Ann. Neurol..

[119] Kitley J., Waters P., Woodhall M., Leite M.I., Murchison A., George J., Kuker W., Chandratre S., Vincent A., Palace J. (2014). Neuromyelitis optica spectrum disorders with aquaporin-4 and myelin-oligodendrocyte glycoprotein antibodies: A comparative study. JAMA Neurol..

[120] Ramanathan S., Reddel S.W., Henderson A., Parratt J.D., Barnett M., Gatt P.N., Merheb V., Kumaran R.Y., Pathmanandavel K., Sinmaz N. (2014). Antibodies to myelin oligodendrocyte glycoprotein in bilateral and recurrent optic neuritis. Neurol. Neuroimmunol. Neuroinflamm..

[121] Hoftberger R., Sepulveda M., Armangue T., Blanco Y., Rostasy K., Calvo A.C., Olascoaga J., Ramio-Torrenta L., Reindl M., Benito-Leon J. (2015). Antibodies to mog and aqp4 in adults with neuromyelitis optica and suspected limited forms of the disease. Mult. Scler..

[122] Jarius S., Kleiter I., Ruprecht K., Asgari N., Pitarokoili K., Borisow N., Hummert M.W., Trebst C., Pache F., Winkelmann A. (2016). Mog-igg in nmo and related disorders: A multicenter study of 50 patients. Part 3: Brainstem involvement—Frequency, presentation and outcome. J. Neuroinflamm..

[123] Spadaro M., Gerdes L.A., Krumbholz M., Ertl-Wagner B., Thaler F.S., Schuh E., Metz I., Blaschek A., Dick A., Bruck W. (2016). Autoantibodies to mog in a distinct subgroup of adult multiple sclerosis. Neurol. Neuroimmunol. Neuroinflamm..

[124] Ramanathan S., Mohammad S., Tantsis E., Nguyen T.K., Merheb V., Fung V.S.C., White O.B., Broadley S., Lechner-Scott J., Vucic S. (2018). Clinical course, therapeutic responses and outcomes in relapsing mog antibody-associated demyelination. J. Neurol. Neurosurg. Psychiatry.

[125] Reindl M., Di Pauli F., Rostasy K., Berger T. (2013). The spectrum of mog autoantibody-associated demyelinating diseases. Nat. Rev. Neurol..

[126] Kerlero de Rosbo N., Honegger P., Lassmann H., Matthieu J.M. (1990). Demyelination induced in aggregating brain cell cultures by a monoclonal antibody against myelin/oligodendrocyte glycoprotein. J. Neurochem..

[127] Piddlesden S.J., Lassmann H., Zimprich F., Morgan B.P., Linington C. (1993). The demyelinating potential of antibodies to myelin oligodendrocyte glycoprotein is related to their ability to fix complement. Am. J. Pathol..

[128] Bettelli E., Baeten D., Jager A., Sobel R.A., Kuchroo V.K. (2006). Myelin oligodendrocyte glycoprotein-specific t and b cells cooperate to induce a devic-like disease in mice. J. Clin. Investig..

[129] Krishnamoorthy G., Lassmann H., Wekerle H., Holz A. (2006). Spontaneous opticospinal encephalomyelitis in a double-transgenic mouse model of autoimmune t cell/b cell cooperation. J. Clin. Investig..

[130] Saadoun S., Waters P., Owens G.P., Bennett J.L., Vincent A., Papadopoulos M.C. (2014). Neuromyelitis optica mog-igg causes reversible lesions in mouse brain. Acta Neuropathol. Commun..

[131] Sato D.K., Callegaro D., Lana-Peixoto M.A., Waters P.J., de Haidar Jorge F.M., Takahashi T., Nakashima I., Apostolos-Pereira S.L., Talim N., Simm R.F. (2014). Distinction between mog antibody-positive and aqp4 antibody-positive nmo spectrum disorders. Neurology.

[132] Jarius S., Ruprecht K., Kleiter I., Borisow N., Asgari N., Pitarokoili K., Pache F., Stich O., Beume L.A., Hummert M.W. (2016). Mog-igg in nmo and related disorders: A multicenter study of 50 patients. Part 2: Epidemiology, clinical presentation, radiological and laboratory features, treatment responses, and long-term outcome. J. Neuroinflamm..

[133] Jarius S., Paul F., Aktas O., Asgari N., Dale R.C., de Seze J., Franciotta D., Fujihara K., Jacob A., Kim H.J. (2018). Mog encephalomyelitis: International recommendations on diagnosis and antibody testing. J. Neuroinflamm..

[134] Chalmoukou K., Alexopoulos H., Akrivou S., Stathopoulos P., Reindl M., Dalakas M.C. (2015). Anti-mog antibodies are frequently associated with steroid-sensitive recurrent optic neuritis. Neurol. Neuroimmunol. Neuroinflamm..

